# Pancreatic Cancer Surgery: What Matters to Patients?

**DOI:** 10.3390/jcm12144611

**Published:** 2023-07-11

**Authors:** David Martin, Piero Alberti, Stephen J. Wigmore, Nicolas Demartines, Gaëtan-Romain Joliat

**Affiliations:** 1 Department of Visceral Surgery, University Hospital CHUV, University of Lausanne (UNIL), 1005 Lausanne, Switzerland; demartines@chuv.ch (N.D.); gaetan-romain.joliat@chuv.ch (G.-R.J.); 2 Department of Surgery, Hepatobiliary and Pancreatic Unit, Royal Infirmary of Edinburgh, Edinburgh EH16 4SA, UK; piero.albertidelgado@nhs.scot (P.A.); s.wigmore@ed.ac.uk (S.J.W.)

**Keywords:** pancreatic cancer, patient-reported outcome measures, patient-reported experience measures

## Abstract

Pancreatic cancer is a leading cause of cancer-related death, with a poor overall survival rate. Although certain risk factors have been identified, the origins of pancreatic cancer are still not fully understood. Surgical resection remains the primary curative treatment, but pancreatic surgery is still associated with high morbidity and mortality rates, and most patients will experience recurrence. The impact of pancreatic cancer on patients’ quality of life is significant, with an important loss of healthy life in affected individuals. Traditional outcome parameters, such as length of hospital stay, do not fully capture what matters to patients during recovery. Patient-centered care is therefore central, and the patient’s perspective should be considered in pre-operative discussions. Patient-reported outcome and experience measures (PROMs and PREMs) could play an important role in assessing patient perspectives, but standardized methodology for evaluating and reporting them is needed. This narrative review aims to provide a comprehensive overview of patient perspectives and different patient-reported measures in pancreatic cancer surgery. Understanding the patient perspective is crucial for delivering patient-centered care and improving outcomes for patients with pancreatic cancer.

## 1. Introduction

Pancreatic cancer (PC) represents the fourth leading cause of cancer-related death, and has been estimated to become the second by 2030 [1,2]. An overall five-year survival rate of 19% has been reported in patients with histologically confirmed PC undergoing surgical resection [3]. The origins of PC are still poorly understood, although certain risk factors have been identified, like smoking, obesity, genetics, diabetes, poor diet and physical inactivity [4]. There are many different systems utilized for the staging of PC. One of the most applied systems that define resectability by analyzing the extent of vascular involvement is the National Comprehensive Cancer Network (NCCN) guidelines [5,6]. The NCCN criteria determine resectability status as three different scenarios: resectable; borderline resectable; and unresectable. They are suggested as a valuable tool in aiding medical and surgical decision-making strategies. Surgical resection remains the primary curative treatment for patients with PC, although only 20% will present with initially resectable disease [1,7]. In a prospective survey of patients with resectable tumors of the head of the pancreas, surgery was preferred by the vast majority (97%), and the main reason was the hope of being cured [8]. PC surgery, however, is associated with high post-operative morbidity and mortality rates, and even after surgical resection, most patients have recurrence of their cancer [9].

PC therefore has particularities in terms of surgical treatment, complications and poor survival, which inevitably impacts patients’ quality of life. A systematic review of the burden of PC in Europe demonstrated a 98% loss of healthy life in affected individuals [10]. Traditionally, research and investigations focused on clinical objective measures, such as length of hospital stay, complication rates, mortality, survival and costs. Although these clinical parameters are relevant to health care professionals and system-level stakeholders, such as hospital administrators and funders, they do not reflect the complexity of the recovery process and the perspective of patients, which are also fundamental [11]. Patient-centered care is defined by the delivery of care that is respectful of and responsive to individual patient preferences, needs and values [12]. This seems particularly important in patients with PC due to the often limited survival after morbid and complex surgeries requiring careful appraisal and shared decision-making [13].

Patients should thus have the opportunity to express their expectations and experiences for the care that they receive. There has been growing interest in this approach for a decade, and this could be beneficial to patients, families and care providers themselves [14,15]. These viewpoints, however, are still not regularly investigated in daily clinical practice. To address this concern and extend this care approach more comprehensively, the meaning of recovery from the patient’s point of view must first be defined.

This scoping review aims to provide a comprehensive overview of patient perspectives, and provide a summary of the different patient-reported outcome and experience measures of patients undergoing pancreatic surgery for PC.

## 2. Patient-Reported Outcome Measures (PROMs) in Pancreatic Cancer Surgery

A patient’s definition of a successful outcome may be different from that of surgeons. Patient-reported outcome measures (PROMs) are defined as any patient’s health condition self-report without interpretation of a response by a caregiver [16,17]. PROMs go beyond the traditional clinical and oncological measures, and assess perceptions of the patient’s health status, perceived impairment, disability and quality of life [15]. Few of the PROMs have been developed, validated and tested for reliability, specifically in patients with PC [18]. Additionally, patients with resectable, borderline resectable and unresectable PC are often mixed within the cohorts studied. In a systematic review assessing PROMs in pancreatic cancer, when specified, only a quarter (24%) of the studies were conducted in patients with resectable PC (15% not specified, 14 all stages mixed) [18]. This therefore limits comparisons between different groups of patients.

A prospective qualitative study including patients undergoing abdominal surgery showed that the themes of recovery identified were returning to habits and routines, resolution of symptoms, overcoming mental strains, regaining independence and enjoying life [11]. Interestingly, the traditional outcomes (hospital stay, complications, costs) were not attributed to a successful recovery by the patients themselves. A multicenter Delphi study among 501 patients with PC in curative and palliative settings identified 8 PROMs that reached a consensus: general quality of life; general health; physical ability; ability to work/do usual activities; fear of recurrence; satisfaction with services/care organization; abdominal complaints; and relationship with partner/family [13]. In a previous Dutch qualitative study, a similar core set of PROMs was selected by patients with PC [19]. These PROMs include general domains, such as quality of life and health, but also psychosocial domains, which have traditionally been understudied [20]. Interestingly, some PROMs with diagnostic and treatment-related importance such as vomiting, jaundice and itching did not reach a consensus among patients [13,19]. Other specific physical domain PROMs, such as sexuality, dizziness, limitation in amount of food tolerated, dysphagia and enteral nutrition, for example, have been described as having low priority.

PROMs usually take the form of questionnaires that can be completed pre- and post-operatively, allowing a better understanding of the recovery process [21]. Disease-specific PROMs are usually more sensitive in capturing changes than generic PROMs [22]. However, assessing PROMs in PC has unfortunately been rarely reported, and no tool has been clearly validated and systematically applied in PC research, thus limiting interpretations and comparisons. Additionally, patients with resectable, borderline resectable and unresectable PC are often mixed within the cohorts studied. In a systematic review assessing PROMs in pancreatic cancer, when specified, only a quarter (24%) of the studies were conducted in patients with resectable PC (15% not specified, 14 all stages mixed) [18]. A disease-specific questionnaire module to PC to supplement the EORTC core cancer module (QLQ-C30) was developed in the late 90s [23]. The resulting module, the QLQ-PAN26, includes 26 items related to symptoms, treatment side effects and emotional issues specific to PC. However, the PAN26 is complex and takes time to complete, and only a limited number of studies describing data collected with it, and specifically validating it, have been published [24].

Another brief, fully patient-derived and disease-specific questionnaire has been developed by a Norvegian team [25]. This PAncreatic CAncer Disease Impact (PACADI) score has been validated across 210 patients with PC, and could significantly predict mortality within the first year in contrast to other questionnaires [22].

The Functional Assessment of Cancer Therapy-Hepatobiliary (FACT-HEP) is a questionnaire measuring general and hepatobiliary disease specific aspects of quality of life, including 27 core items and an additional 18 hepatobiliary-specific items (5-point Likert-type scales) that assess symptoms and function [26,27]. This questionnaire has been specifically validated in patients with metastatic PC [28]. However, the FACT-HEP is long to complete and complex to interpret. Furthermore, compliance, as well as the response rate to the questionnaires remain to be investigated.

The M.D. Anderson Symptom Inventory for gastrointestinal cancer (MDASI-GI) was developed to assess physical and psychological symptom prevalence and burden among the GI cancer population [29]. It contains 24 items to be evaluated on a scale of 1 (“not present”) to 10 (“as bad as you can imagine”). It has been validated in a group of 46 patients with PC [29].

The Gastrointestinal Quality of Life Index (GIQLI) is an instrument designed to measure the quality of life in patients with gastrointestinal disease [30]. It measures the subjective perception of well-being by the patient, and consists of 36 items encompassing gastrointestinal symptoms, psychological state, physical function and social function. A high score indicates a better quality of life (maximum score 144). The GIGLI has been used in patients undergoing pancreaticoduodenectomies for cancer in 2 studies, with respective mean scores of 120 and 118 [30,31].

Patients with PC often suffer from disease-related symptoms. The Edmonton Symptom Assessment System (ESAS) measures the severity of 9-symptom domains and has been validated for use in oncology, including PC [32]. A population-based cohort study using the ESAS following curative intent pancreaticoduodenectomy showed that the proportion of patients with moderate to severe symptoms was highest immediately after surgery and decreased over time, stabilizing at around 3 months [33].

The Hospital and Anxiety Depression Scale (HADS) is a self-rating scale, mainly used for rapid assessment of patients’ anxiety and depression, and is one of the tools for the screening of these psychological aspects [34,35,36]. The scale has 14 items, including 7 items for anxiety and 7 items for depression. Its validity has been demonstrated in different types of cancer surgery, including PC [37,38,39,40].

The Acceptance of Illness Scale (AIS) is a questionnaire designed for measuring any disease acceptance [41]. This tool is constituted of 8 statements forming a single scale, each graded from 1 to 5, where 1 stands for “I strongly agree” and 5 for “I strongly disagree”. The total score ranges between 8 and 40 points, which will reflect the degree of illness acceptance. A low AIS score shows no acceptance of the condition and mental discomfort, while a high score is indicative of good disease acceptance [41]. The mean score for patients with PC is 23, which is the lowest score among gastrointestinal cancers [42].

The Katz Activities of Daily Living (ADL) index is a dichotomous categorization of 6 daily activities: bathing; dressing; toileting; transferring; continence; and feeding. Scores range from 6 (independence in all activities of daily living) to 0 (dependence in all 6 activities of daily living) [43]. A study using Medicare Current Beneficiary Survey (MCBS) data showed that the mean score significantly increased from 0.5 pre-diagnosis to 1.5 post-diagnosis (*p* = 0.0015) of PC [44].

A systematic review confirmed that PROMs currently used in abdominal surgery lack adequate measurement properties, which precludes their use to support value-based surgical care [45]. Investigations of PROMs are typically based on input from physicians with only limited scientific methodology, or originating from single countries, limiting international generalizability [13]. Another systematic review showed that the median completion rates of PROM questionnaires fell to below 10% in patients with unresectable PC, compared to 75% in those with resectable PC [18]. Furthermore, due to social-cultural differences, priorities and expectations of patients may differ between various regions, and this also might influence the selection of PROMs and the interpretation of the results [19,46].

Finally, the Pancreatic Cancer Action Network (PanCAN) patient registry, developed by an American team, is an online, voluntary pancreatic cancer-specific registry enabling patients to self-report sociodemographics, disease/management characteristics and PROMs [47]. This type of registry can facilitate standardized PROMs reporting and monitoring from patients worldwide, and provide a valuable research database.

## 3. Patient-Reported Experience Measures (PREMs) in Pancreatic Cancer Surgery

Patient-reported experience measures (PREMs) are tools for assessing experiences with health care from the patient’s perspective. While PROMs measure health care outcomes as described above (e.g., symptoms or health-related quality of life), PREMs assess patients’ needs and experiences of their care or health service (e.g., involvement in care decision-making or accessibility of services) [15,48]. Table 1 provides a summary of described PROMs and PREMs in patients with PC. It has been shown that high levels of positive patient experiences are associated with higher levels of adherence to recommended treatment processes and better clinical outcomes [49,50]. Compared to PROMs that have been described and used for PC patients, the application of PREMs is rather scant in oncological surgery, especially in pancreatic surgery. Indeed, there remains some reluctance regarding their use, probably due to some of their limitations. PREMs may be seen as similar with terms such as “patient satisfaction” and “patient expectation”, both of which are subjective terms that can be reflective of judgments on the adequacy of health care rather than quality [51]. PREMs may also be biased by factors not directly related to the quality of health care experienced by patients, and could be a reflection of patients’ preconceived health care expectations and not their actual care experience [51,52]. Despite these limitations, PREMs can be used for clinical research, quality improvement projects, clinician performance assessment (audit) and economic evaluation. Furthermore, they can also be utilized as a common measure for public reporting, the benchmarking of institutions, and more generally, health care plans [52].

A systematic review concluded that PREMs with good psychometric characteristics were lacking in oncology, and had low reliability and construct validity [53]. Another review concluded that cancer patients treated in hospital in curative intent found information about treatment and consequences, professional standards, as well as short delay of diagnosis and treatment most important [54]. Specifically for PC, a cross-sectional questionnaire survey of patients in UK revealed that 29% of respondents did not receive enough information at diagnosis, and 10% felt that they were not involved in decisions about their treatment, but would have liked to be [55].

The Picker Patient Experience (PPE) questionnaire is a tool for assessing the patient experience of care, and consists of 15 questions assessing eight key aspects of patient care [56]. These aspects include information and education, coordination of care, physical comfort, emotional support, respect for patient preferences, involvement of family and friends, continuity and transition and overall impression. The questions are designed with a range of possible responses, which are later converted into a binary outcome indicating whether there was a problem or not in the questioned domain. By analyzing these binary responses, the percentage of patients who experienced problems or not can be calculated for each question. The validation process involved the administration of the PPE questionnaire to inpatients in five countries (Australia, Canada, New Zealand, the United Kingdom and the United States) [56]. The analysis showed that the PPE questionnaire had good psychometric properties, including high internal consistency and reliability. Additionally, the questionnaire was found to be sensitive to differences in patient experiences of care across countries, emphasizing the importance of cultural and contextual factors in shaping patient experience. The authors concluded that the PPE questionnaire was a valid and reliable tool for assessing the patient experience of care across different healthcare systems and cultural contexts, and that it could be used to assess quality improvement efforts and benchmarking across healthcare facilities and countries.

The EORTC IN-PATSAT32 questionnaire is a tool evaluating patient satisfaction with care during hospital stays [57]. The questionnaire includes 32 questions that evaluate patient satisfaction with doctors, nurses, services, care organization and care in general. Patients are asked to rate their satisfaction on a 5-point Likert scale ranging from 1 (poor) to 5 (excellent). The questionnaire covers various aspects of care, including communication, coordination of care, quality of medical treatment and physical comfort. The EORTC IN-PATSAT32 questionnaire can be used to identify areas of care that need improvement, and to evaluate the impact of quality improvement initiatives on patient satisfaction. It has been tested in patients before and after treatment of pancreatic and periampullary cancer [58]. General satisfaction with care decreased after treatment, highlighting the need for improvements in communication and interpersonal skills.

The Nordic Patient Experiences Questionnaire (NORPEQ) is a short self-completed questionnaire with evidence for data quality, reliability and validity [59]. The NORPEQ includes what are judged to be the most important aspects of experiences for patients, including the understanding, competence and interest of doctors and nursing staff, as well as the information delivered on tests/examinations, relevance of treatment and overall care satisfaction [60]. However, it has not been validated in patients with PC.

The patient experience questionnaire (PEQ) was produced with 18 items on five dimensions (communication, emotions, outcome, barriers and relations with the staff) [61]. The validity and reliability of the questionnaire were satisfactory, and it highlights what matters the most to patients, and thus may represent a valuable tool for doctors who want feedback from their patients on the function of their doctor-patient relationships [61].

Table 2 summarizes the available tools for PROMs and PREMs in pancreatic cancer surgery.

## 4. Discussion

The majority of PROM and PREM questionnaires are given to patients at different key moments in patient care, typically in pre-operative clinics with the post-operative questionnaire being collected after surgery. The ideal timing to complete the questionnaire may vary depending on the type of procedure and associated additional treatments (chemotherapy, recovery, etc). There has been an increase in the number of electronic tools developed to support patients during cancer care. Patient portals and groups on social media with various and different means of virtual communications did appear. A systematic review showed that electronic systems have the potential to help patients manage side effects of cancer treatment, though comparison across studies was difficult due to the wide range of available assessment tools [66]. Structured interviews have also been suggested to assess patient perspectives, but these are complex and time-consuming and require training and resources. Recent European Society for Medical Oncology (ESMO) guidelines recommend digital symptom monitoring in routine clinical care during cancer treatment, with the use of web-based platforms and smartphone applications which can be accessed by electronic devices [16]. Patient registry might be a good option, insofar as patients can have free access to the platform, fill in the data when they have the time and the motivation and give feedback and opinions in the form of free text. In the PanCAN registry, it is important to note that 95% of patients want to provide information for researchers and other patients, and 90% want to learn more about pancreatic cancer [47].

To avoid patient burden and increase patient compliance, tools and items should be carefully selected. It should also be emphasized that the more often a questionnaire is administered to patients, the shorter it should be. For example, several successful studies have adopted only 10 to 20 items for weekly administration [14,67]. Furthermore, whenever possible, more than one mode of administration (paper-based, emails, phone calls…) should be offered, to ensure that vulnerable populations can also have access to it [16]. It has also been suggested that completing the questionnaires at home, during the patient’s own time, might contribute to increased compliance and completion rates [68]. A probably often neglected aspect is the verification of the literacy of patients, and that the language of questionnaires is appropriate for the general population. The reading ability in the general public might be lower than healthcare professionals think.

**Figure 1:**
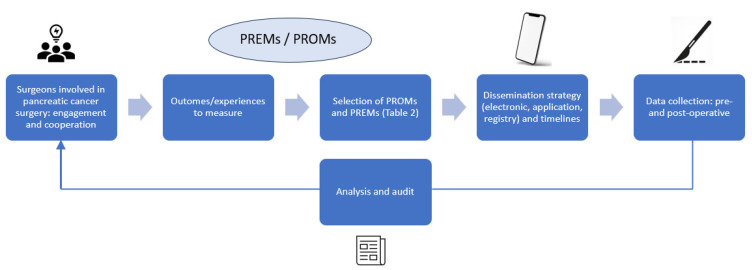
Steps for integrating PROMs and PREMs in clinical practice.

Several limitations need to be considered when collecting and interpreting PROM and PREM data. The patients may not be in a physical or psychological state to give accurate feedback. They may also be anxious about the potential negative impact of their answers on the management of their pathology, thus adjusting their responses accordingly by overrating healthcare quality [68]. Time constraints from the involved staff may also affect data collection process. This may induce some inadvertent selection of patients depending on the available resources. Routine implementation of these tools represents a real challenge, and there remain issues to be investigated: cancer-specific questionnaires; method of delivery; software systems; timing/frequency of distribution; number of reminders; minimum response rates; and privacy/security of the collected data. Future clinical trials and research should be include PROMs and PREMS in a systematic way to measure the success of the treatments and the outcomes. This could be done by establishing national and international consensus guidelines, during congresses and/or other meetings, with help and promotion by surgical societies in close collaboration with oncological societies. Patient-completed registries are also an option for further investment, but their dissemination and validity of data remain important issues to clarify and study in the future. For clinical practice, the development and implementation of a PROMs and PREMs governance framework is suggested in Figure 1.

## 5. Conclusions

Traditional outcome parameters do not fully capture what matters to patients recovering from PC surgery. Patients’ perspectives include an interplay of physical, psychological and social factors that should be addressed in pre-operative discussions when planning surgery for pancreatic cancer. PROMs and PREMs will play an important role in the near future, but there is a crucial need to develop standardized methodology for assessing and reporting them.

## Figures and Tables

**Table 1:** Patient-reported outcome and experience measures.

**PROMs**					
	**Work/Routines**	**Health/Symptoms**	**Mental Issues**	**Independence**	**Quality of Life**
	- Physical activity and sports - Household responsibilities - Relationship with family - Social life - Ability to work	- Eating habits - Pain - Bowel movements - Nausea/vomiting - Surgical scars - Difficulty sleeping - Weakness - Itchiness	- Fatigue - Depression - Concerns about the procedure’s success - Fear of recurrence - Reaching full recovery - Life changes after surgery	- Personal hygiene - Moving around, driving - Activities of daily living	- Having fun - Traveling - Hobbies (sport, games, reading…) - Adventures
**Tools**	QLQ-PAN26, FACT-HEP	PAN26, PACADI FACT-HEP, ESAS, MDASI-GI	PAN26, PACADI, FACT-HEP, AIS, MDASI-GI, HADS	Katz index	QLQ-PAN26
**PREMs**					
	**Professional Standards**	**Diagnosis/Information**	**Organization**	**Decisions**	**Support**
	- Conscientious work - Caring caregivers	- Short delay - Clear communication - Transparency - Treatment consequences	- Continuity among caregivers - Coordination of care	- Active participation	- Empathy - Respect - Psychological support - Easy access to caregivers or hospital - Involvement of family
**Tools**	PPE, IN-PATSAT32, interviews	PPE, IN-PATSAT32, patient portals, social media	PPE, IN-PATSAT32, mobile applications	Interviews	PPE, mobile applications, patient portals

PROMs, Patient-reported outcome measures; PREMs, Patient-reported experience measures; QLQ, Quality of Life Questionnaire; PACADI, Pancreatic Cancer disease impact; FACT-HEP, Functional Assessment of Cancer Therapy-Hepatobiliary; ESAS, Edmonton Symptom Assessment System; MDASI-GI, MD Anderson Symptom Inventory for gastrointestinal cancer; HADS, Hospital Anxiety and Depression Scale; AIS, Acceptance of Illness Scale; PPE, Picker Patient Experience; IN-PATSAT32, Inpatients’ Satisfaction 32.

**Table 2:** Key instruments for PROMs and PREMs available for use in pancreatic cancer surgery.

	Study/Year	Year	Instrument	Items	Scope	Validation Cohort
**PROMs**	Katz et al. [43]	1970	Katz index	8	Daily activities: bathing, dressing, toileting, transferring, continence, feeding	All stages of PC [44]
	Zigmond et al. [36]	1983	HADS	14	Anxiety and depression	All stages of PC [40]
	Felton et al. [62]	1984	AIS	8	Disease acceptance	All stages of PC [42]
	Bruera et al. [63]	1991	ESAS	10	Pain, tiredness, nausea, depression, anxiety, drowsiness, appetite, well-being, shortness of breath	Resectable PC [33]
	Fitzsimmons et al. [23]	1997	QLQ-PAN26	26	Functional, physical, emotional, social, cognitive	Resectable PC [24]
	Eypasch et al. [64]	1995	GIQLI	36	Physical, emotional, social, symptoms	Resectable PC [30,31]
	Cleeland et al. [65]	2000	MDASI-GI	24	Physical/psychological symptoms	All stages of PC [29]
	Yount et al. [26]	2002	FACT-HEP	45	Quality of life, symptoms and function	Unresectable PC [28]
	Heiberg et al. [25]	2013	PACADI	8	Pain/discomfort, fatigue, anxiety, digestive problems, loss of appetite, dry mouth, itchiness, nausea	All stages of PC [22]
**PREMS**	Steine et al. [61]	2001	PEQ	18	Communication, emotions, outcome, barriers, relations with staff	Not specifically for PC [61]
	Jenkinson et al. [56]	2002	PPE	15	Information/education, coordination of care, physical comfort, emotional support, respect for patient preferences, involvement of family/friends, continuity and transition, overall impression	Not specifically for PC [56]
	Brédart et al. [57]	2005	IN-PATSAT32	32	Satisfaction with doctors, nurses, services, care organization. Covers various aspects: communication, coordination of care, quality of medical treatment, physical comfort	All stages of PC [58]
	Oltedal et al. [60]	2007	NORPEQ	8	Doctors/nursing staff understanding, competence, interest, information delivered on tests/examinations, care satisfaction, relevance of treatment	Not specifically for PC [59]

PROMs, Patient-reported outcome measures; PREMs, Patient-reported experience measures; HADS, Hospital Anxiety and Depression Scale; AIS, Acceptance of Illness Scale; ESAS, Edmonton Symptom Assessment System; QLQ, Quality of Life Questionnaire; GIQLI, Gastrointestinal Quality of Life Index; MDASI-GI, MD Anderson Symptom Inventory for gastrointestinal cancer; FACT-HEP, Functional Assessment of Cancer Therapy-Hepatobiliary; PACADI: Pancreatic Cancer disease impact; PEQ, patient experience questionnaire; PPE, Picker Patient Experience; IN-PATSAT32, Inpatients’ Satisfaction 32; NORPEQ, Nordic Patient Experiences Questionnaire.

## Data Availability

Not applicable.
